# Case series: a rare dominant form of β-thalassemia successfully treated by luspatercept

**DOI:** 10.1007/s00277-026-06778-8

**Published:** 2026-01-21

**Authors:** Pierre N. Allard, Andreas E. Kulozik, Joachim B. Kunz

**Affiliations:** Department of Pediatric Oncology, Hematology and Immunology, Hopp-Children’s Cancer Center (KiTZ) Heidelberg, University of Heidelberg, Heidelberg, Germany

**Keywords:** Dominant beta-thalassemia, HBB, Anemia, Elongated beta-globin variant

## Abstract

Beta-thalassemia is typically inherited in an autosomal recessive manner and can result from a wide range of mutations affecting all stages of the gene expression pathway. Nonsense and frameshift mutations usually trigger nonsense-mediated mRNA decay (NMD). In rare cases, however, such mutations can lead to dominant β-thalassemia when NMD is bypassed, allowing the synthesis of truncated β-globin chains with dominant-negative effects. We describe a multigenerational family with a novel heterozygous two–base pair insertion in the *HBB* gene (c.287_288insAC), resulting in a frameshift and a premature stop codon located downstream of the NMD threshold. This mutation causes dominantly inherited β-thalassemia with a broad clinical spectrum, ranging from mild anemia to transfusion dependency. The index patients‘ mother presented with anemia, splenomegaly, iron overload, and ultimately became transfusion-dependent. Her three children, all carriers of the same variant, exhibited variable degrees of anemia; two developed symptoms during adolescence that required pharmacological intervention. Luspatercept was effective in the affected mother and her daughter, improving hemoglobin levels, alleviating symptoms, and reducing transfusion requirements. Hydroxyurea was successfully used in the second daughter to stabilize hemoglobin levels. This case series expands the known spectrum of dominant β-thalassemia mutations and highlights the marked phenotypic variability even within a single family. Our findings support the use of Luspatercept and Hydroxyurea as therapeutic options. Long-term monitoring, including surveillance for complications related to iron overload, is essential for optimal clinical management.

## Introduction

Beta-thalassemia is an inherited hemoglobinopathy predominantly found in the Mediterranean region, large parts of Asia, the Middle East, and Africa [1]. It is characterized by reduced or absent production of β-globin chains. Depending on the residual activity of the *HBB* alleles and on modifying factors *in trans*, β-thalassemia may remain asymptomatic (silent β-thalassemia), cause mild anemia (β-thalassemia minor), or lead to severe anemia that may be transfusion-dependent, often accompanied by iron overload and subsequent organ damage (β-thalassemia intermedia or major). Beta-thalassemia is most commonly caused by point mutations [2] and is usually inherited in an autosomal recessive manner. This recessive inheritance pattern is largely attributable to a messenger RNA quality-control mechanism known as nonsense-mediated decay (NMD). NMD degrades mRNAs harboring premature termination codons, thereby preventing the production of potentially deleterious truncated polypeptides. In β-globin mRNA, stop codons trigger NMD when located upstream of a boundary 54 nucleotides 5′ to the final exon–exon junction. In rare cases, *HBB* mutations generate stop codons located downstream of this boundary, allowing the mRNA to escape NMD and resulting in a dominantly inherited form of β-thalassemia [2]. Here, we report a rare heterozygous two–base pair insertion in the *HBB* gene (codon 97 +AC, c.287_288insAC) that causes a dominantly inherited form of β-thalassemia.

## Case description

### Patient II-7, our three index patients‘ mother

Patient II-7 is a 49-year-old woman who was incidentally diagnosed with anemia at the age of 18, initially attributed to blood loss following an accident. At age 22, she was noted to have splenomegaly and hemolytic anemia (total hemoglobin 8.7 g/dL, reticulocytes 60‰, bilirubin 2.1 mg/dL) but remained asymptomatic. Hemoglobin analysis at that time was non-diagnostic. Bone marrow cytology revealed erythroid hyperplasia with normal granulocytopoiesis and megakaryocytopoiesis. An erythrocyte fragility test showed early onset of hemolysis at 0.52% NaCl but delayed complete hemolysis, findings at that time interpreted as consistent with hereditary spherocytosis. A more specific diagnostic evaluation for hereditary spherocytosis is not available.

At age 28, repeat hemoglobin variant analysis (Table 1) not disturbed by prior transfusion revealed normal HbA₂ (2.9%) but elevated HbF (16.2%), raising suspicion for β-thalassemia intermedia. Molecular analysis of the *HBB* gene identified a novel heterozygous two–base pair (+AC) insertion after codon 97 (c.287_288insAC), predicted to evade nonsense-mediated decay, produce a C-terminally altered β-globin, and result in dominantly inherited β-thalassemia.

The patient had three uncomplicated pregnancies at ages 31, 33, and 39, without transfusion requirements. At age 35, she first noted anemia-related symptoms, including fatigue, pallor, jaundice, and reduced exercise tolerance. Her first red blood cell (RBC) transfusion occurred at age 41, when hemoglobin dropped to 4.5 g/dL during an infection. Liver iron concentration at that time indicated iron overload (FerriScan: 310 mmol/kg; normal: 3–33), prompting initiation of daily oral deferasirox (500 mg suspension). Cardiac MRI showed no myocardial siderosis or ventricular dysfunction. From age 44, she received regular RBC transfusions every five weeks for chronic anemia, fatigue, and reduced exercise tolerance. Concurrently, hydroxyurea was administered (ages 44–48) to induce fetal hemoglobin, which reduced but did not eliminate transfusion needs. Upon availability, Luspatercept was initiated (1 mg/kg every three weeks). The patient reports of an improvement of symptoms and an increase of hemoglobin levels to approximately 11.5 g/dL without the need for transfusions over 18 months.

**Fig. 1:**
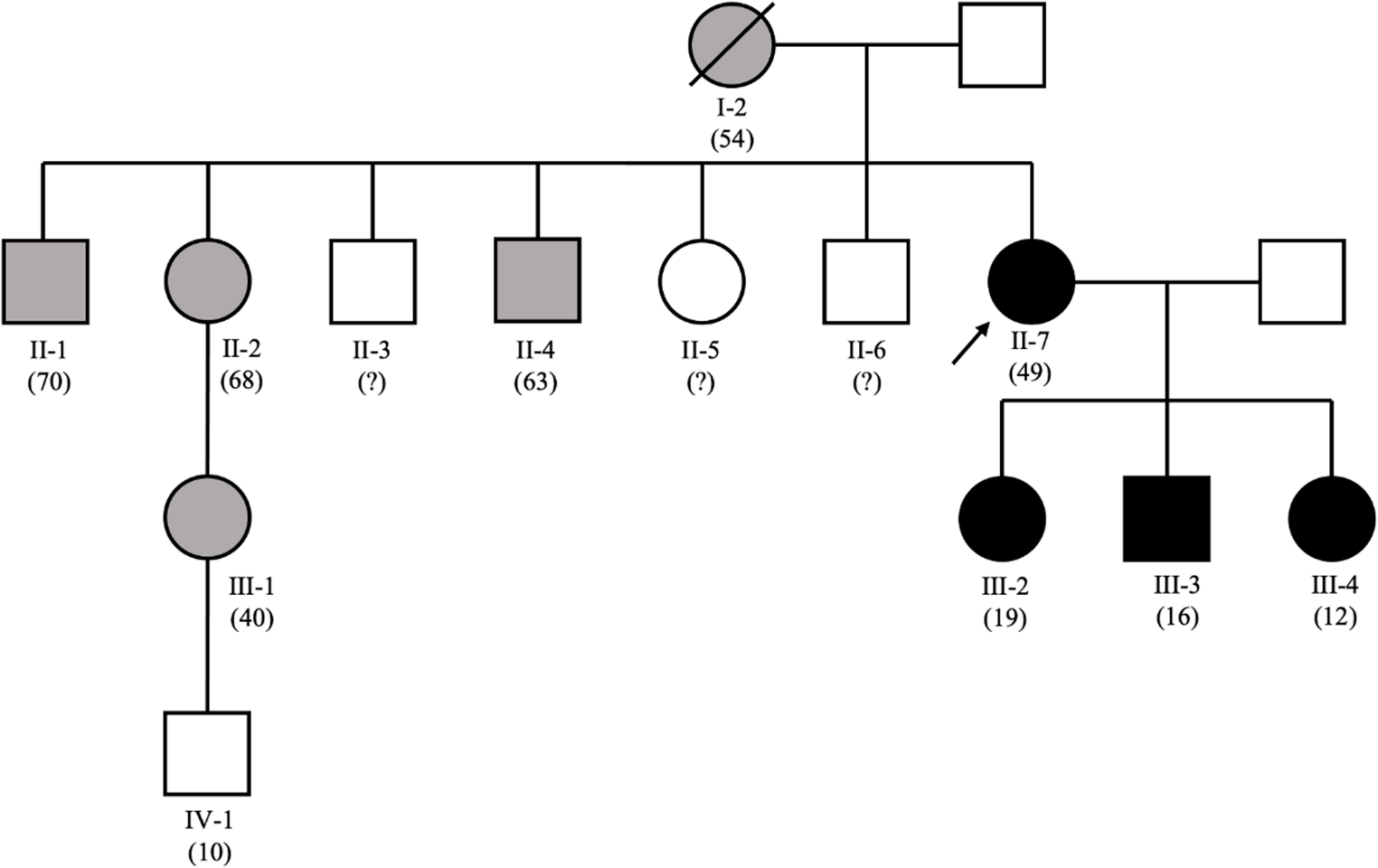
Family pedigree. Index patients are III-2, III-3 and III-4 whose mother is marked by an arrow. Circles and squares respectively indicate the females and males. The relatives in grey did not undergo genetic testing but were affected by symptomatic or transfusion-dependent thalassemia

The patient was born in Izmit, Turkey; her maternal family had migrated from Georgia to Turkey. Most relatives reside in Turkey, and detailed clinical data are unavailable (Fig. 1). Her mother (I-2) had lifelong anemia and jaundice but never received transfusions; she died of cancer at age 54. Her brother (II-1, aged 70) reportedly has splenomegaly and jaundice without transfusion history. Her sister (II-2, aged 68) has marked facial bone prominence; she first required transfusion at age 26 during pregnancy and has received intermittent transfusions since. The sister’s daughter (III-1, aged 40) began regular transfusions at age 37; her 10-year-old son (VI-1) is unaffected. Another brother (II-4, aged 63) has jaundice but has not been tested for thalassemia. Three additional siblings (II-3, II-5, II-6) are reportedly unaffected, though clinical contact is limited. Based on symptoms and transfusion dependency, several relatives likely carry the same *HBB* mutation.

### Index patients

**Fig. 2:**
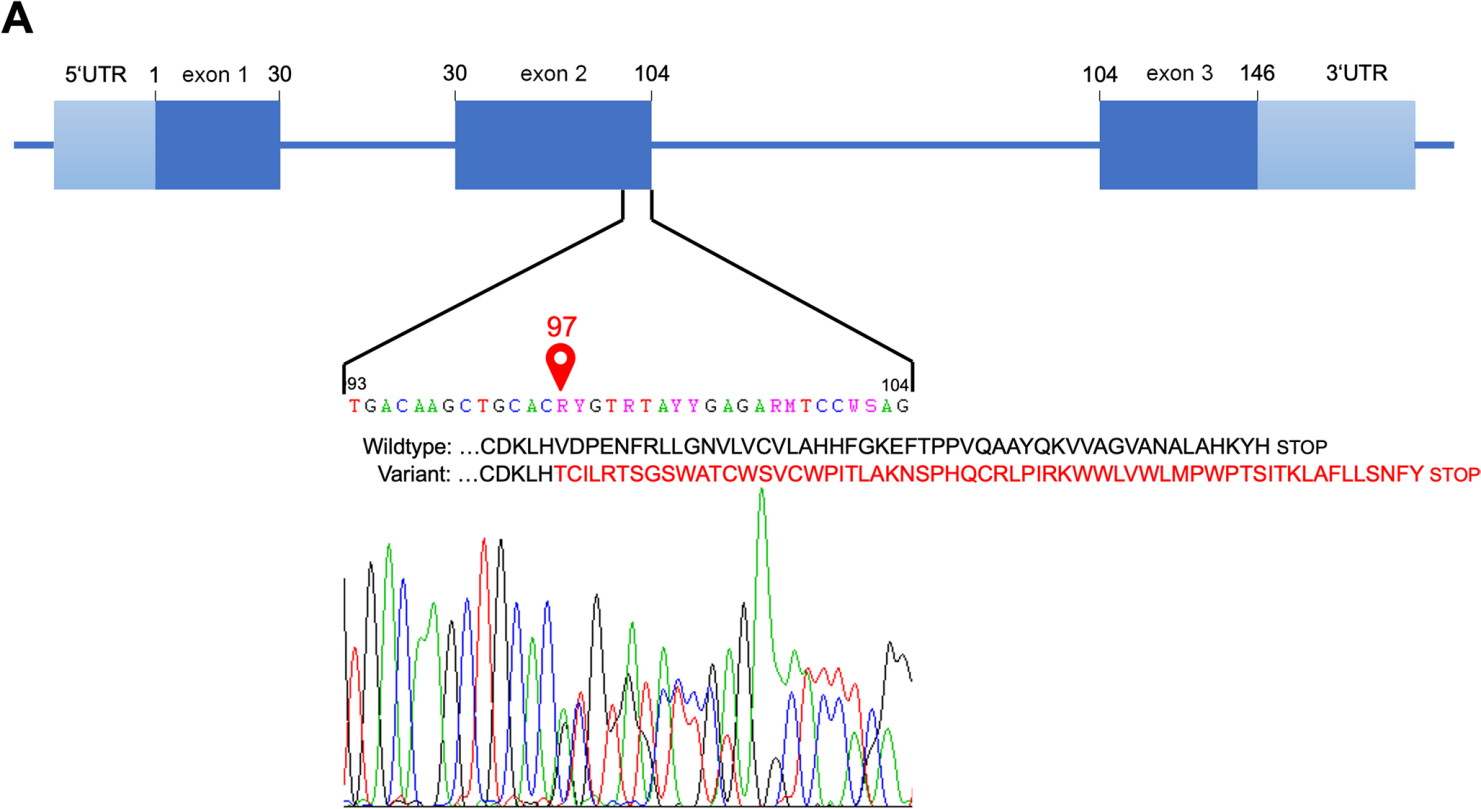

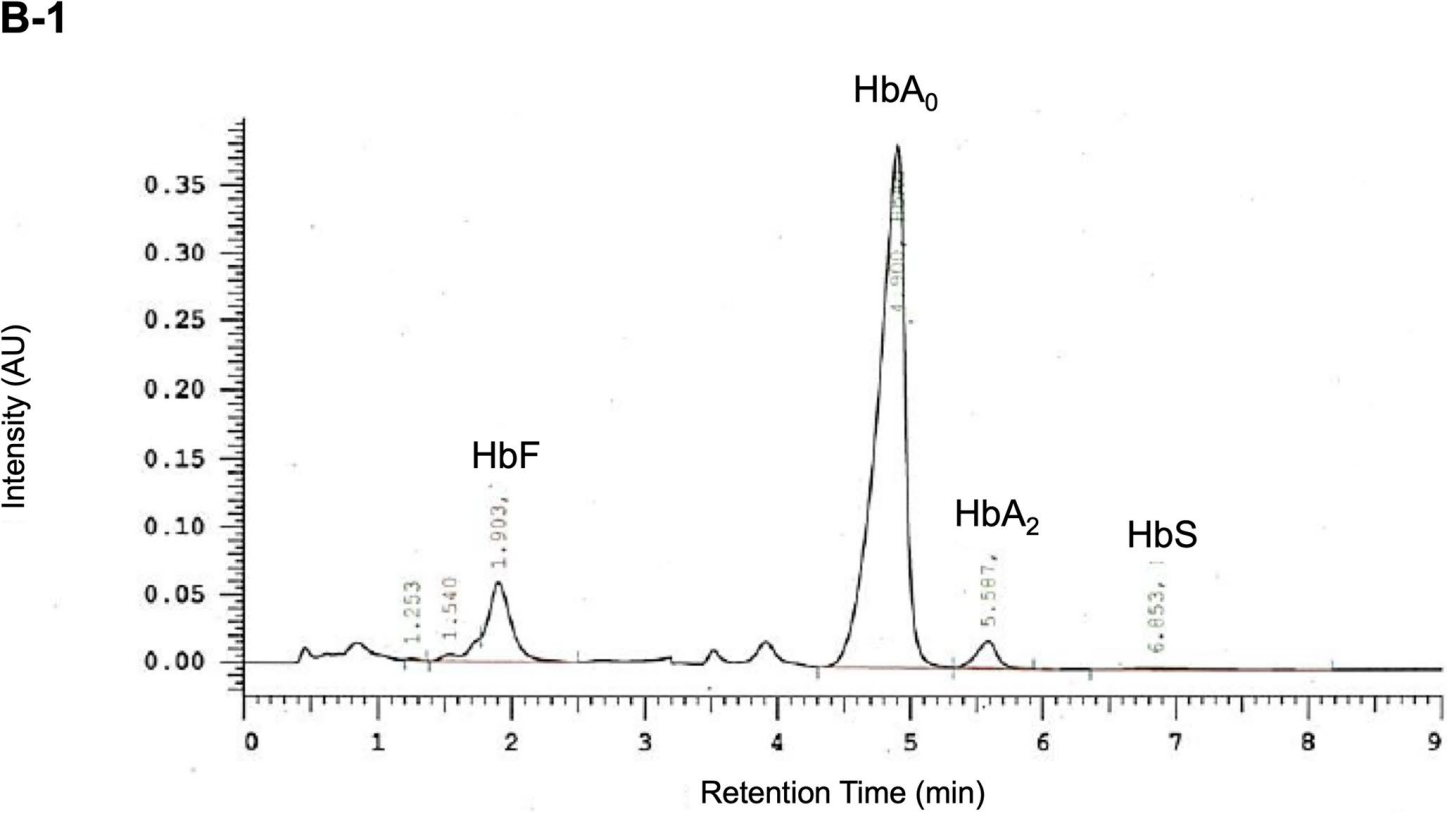

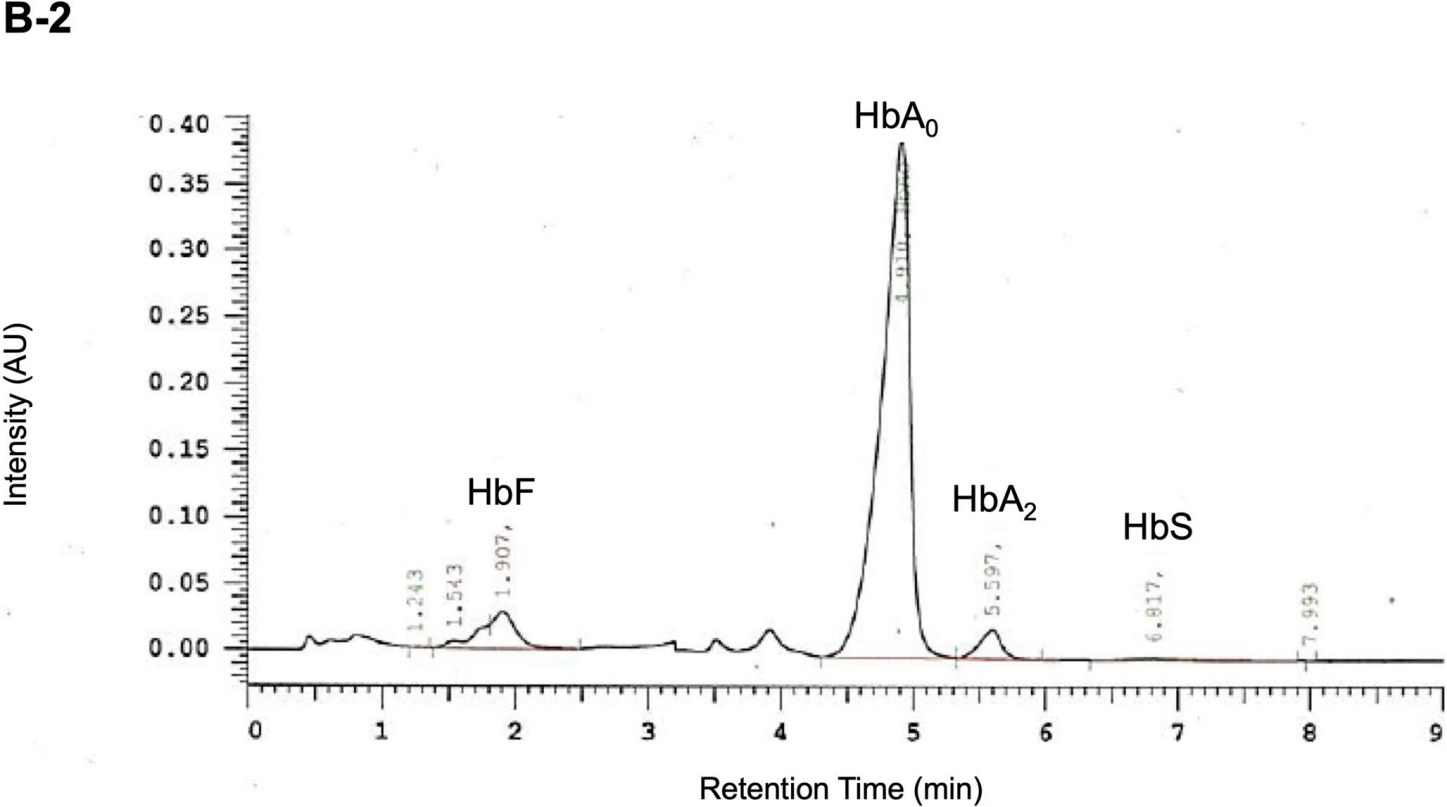
Molecular analysis of *HBB* and Hb-Electrophoresis. **A**: Mutation in codon 97/exon 2 of *HBB* marked red with the surrounding sequence detailed. Dark blue squares represent exons 1 to 3, light blue squares represent 5‘- and 3‘-untranslated regions, dark blue lines represent introns. Amino acid sequence in black for wildtype and in red for the variant. Below: Sanger sequencing electropherogram; **B**: Hb Electropherogram of III-2 (B-1) and III-4 (B-2)

The three children (III-2, III-3, and III-4) exhibited mild anemia-related symptoms, such as pallor and reduced physical capacity, particularly during sports activities. The parents were nonconsanguineous; the father, of German origin, had no history of anemia. In all three children, the same *HBB* c.287_288insAC mutation identified in their mother was confirmed by Sanger sequencing of the β-globin gene (Fig. 2A). This insertion results in a frameshift destroying the physiological stop codon and generates a novel stop codon 3‘ of the original one. The absence of coinherited α-thalassemia was confirmed by sequence-specific PCR for the most common deletions (–α³·⁷, –α⁴·², ––^20.5^, ––^SEA^, ––^MED^) and by sequencing of the α₁- and α₂-globin genes [3]. We tested for known genetic modifiers of fetal hemoglobin production (polymorphisms rs7482144 in *HBG2*, rs66650371 in *HMIP*, rs1427407 and rs7606173 in *BCL11A*) as described earlier [4–6]. All three children were heterozygous for *BCL11A* rs7606173 and the *HBG2* polymorphism rs7482144, both of which are associated with elevated HbF levels [6, 7].

###  Eldest daughter (III-2)

**Fig. 3:**
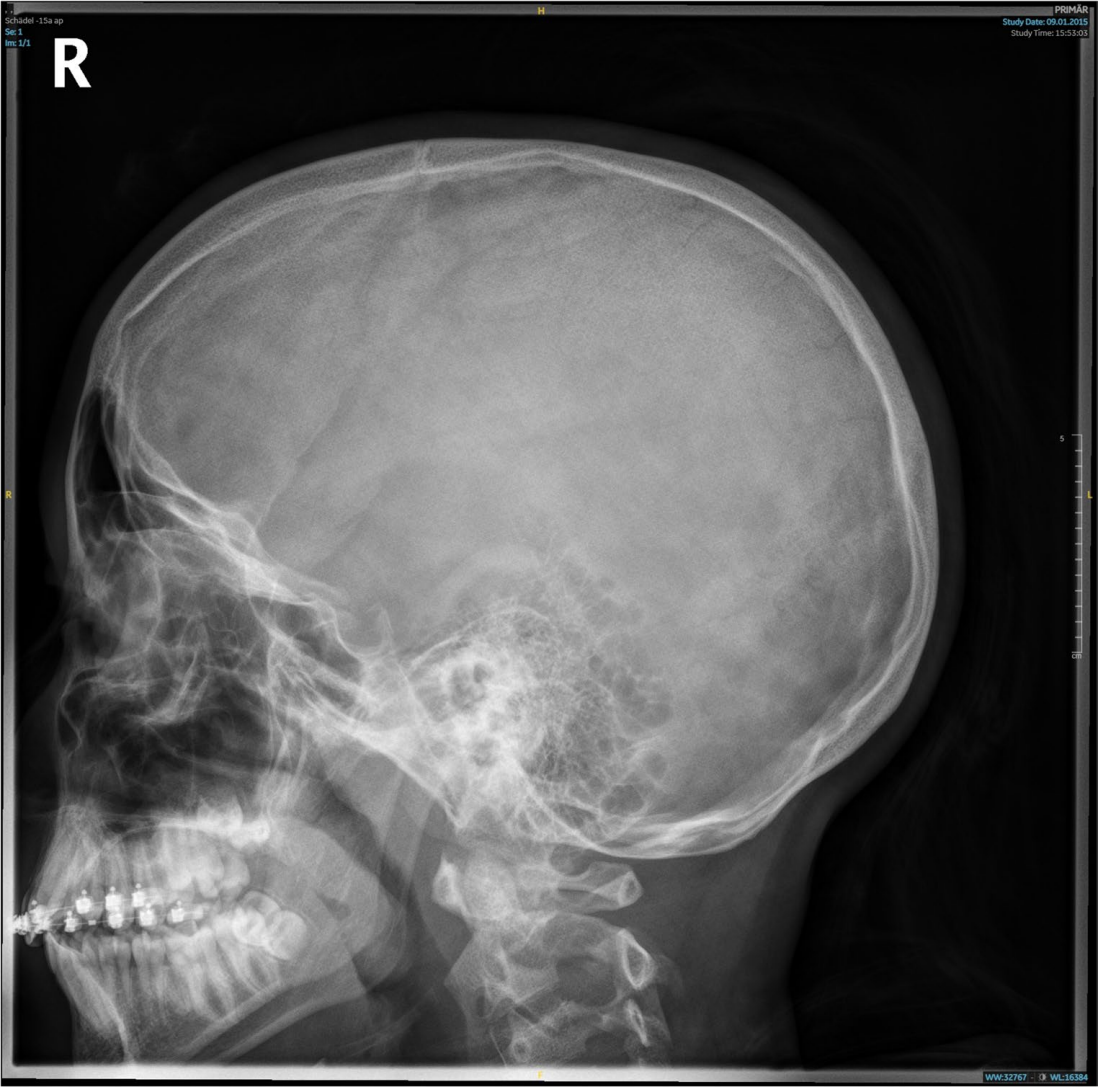
Skull X-Ray of III-2

**Fig. 4:**
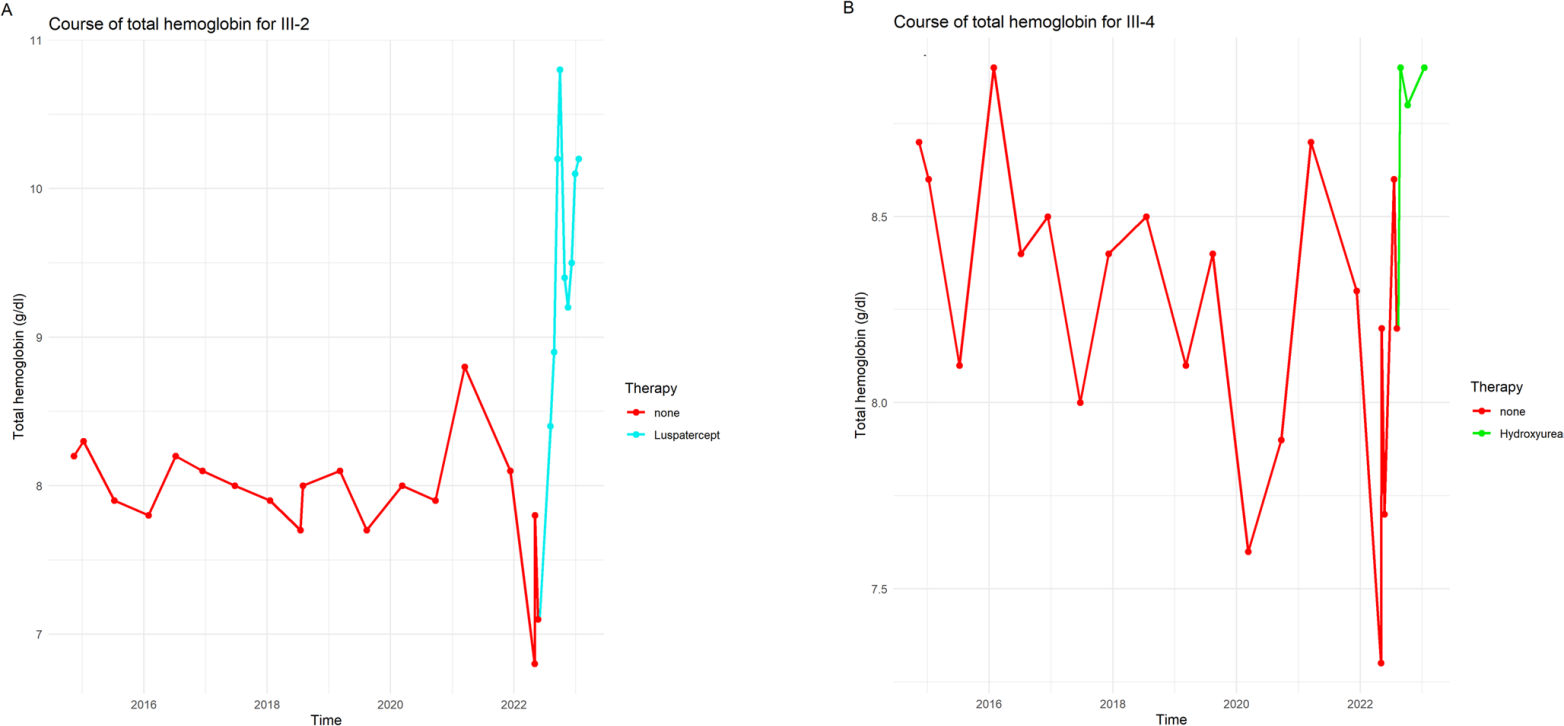
Course of total hemoglobin in III-2 and III-4. **A**: Proband III-2, blue curve under treatment with Luspatercept; **B**: Proband III-4, green curve under treatment with Hydroxyurea

First evaluated at age 11, she had palpable splenomegaly (2 cm below the costal margin) and mild hypochromic microcytic anemia (total hemoglobin 8–9 g/dL; Table 1) with elevated HbF and HbA₂ (Table 1; Fig. 2B). She was well adapted to anemia without daily activity restrictions. Facial features were normal, though skull X-ray revealed mild diploic thickening (Fig. 3) [3]. Liver iron was normal, and abdominal MRI confirmed splenomegaly (length 16.6 cm). She was monitored biannually for signs of extramedullary erythropoiesis and transfusion needs, showing overall stability until age 19, when mild iron overload (2.7 mg/g dry tissue) was detected. At age 18, she experienced hemoglobin decline to 6.8 g/dL with marked fatigue, dizziness, presyncope, and recurrent headaches. Meeting criteria for in-label Luspatercept therapy, she began treatment (1 mg/kg every three weeks) in August 2022. The drug was well tolerated, symptoms resolved, and total hemoglobin rose to 10.8 g/dL by September 2022 (Fig. 4A).

### Son (III-3)

First seen at age 9, he had mild hypochromic microcytic anemia and palpable splenomegaly (Table 1) without activity limitation. Liver iron and skull X-ray were normal. He remains stable with milder anemia symptoms than his sisters and has not required treatment.

### Youngest daughter (III-4)

First seen at age 3, she had mild hypochromic microcytic anemia (Table 1) without splenomegaly or skull abnormalities. Splenomegaly became detectable (2 cm below costal margin) at age 10. At age 11, she showed mild iron overload (2.1 mg/g dry tissue) and total hemoglobin decline to 7.3 g/dL, accompanied by fatigue and headaches. Hydroxyurea (10 mg/kg/day) was initiated in August 2022, preventing transfusion and stabilizing hemoglobin around 8.9 g/dL, with symptomatic improvement (Fig. 4B).

None of the children exhibited growth disturbance per Redlefsen et al. [4] (height/weight percentile: III-2 77th /80th ; III-3 70th /64th ; III-4 39th /37th ).

**Table 1:** Hematological parameters and genotype of the family

Parameters	II-7	III-2		III-3		III-4	
Sex – Age (years)	F – 41	F – 11	F – 19	M – 9	M – 15	F – 3	F – 12
Therapy	None	None	Lusp.	None	None	None	HU
Hb (g/dL) *Reference intervals *[5]	7.5 *(11.4–15.1)*	8.3 *(11.8–15)*	9.1 *(11.4–15.1)*	8.9 *(11.5–14.8)*	10 *(12.5–16.5)*	8.7 *(10.5–13.9)*	7.8 *(11.8–15)*
RBC (/pL) *Reference intervals* [5]	3.3 *(3.9–5.3)*	3.9 *(4.1–5.3)*	4.1 *(3.9–5.3)*	4.8 *(4.2–5.3)*	4.7 *(4.3–5.8)*	4.2 *(3.9–5.3)*	3.8 *(4.1–5.3)*
MCV (fL) *Reference intervals *[5]	68.4 *(79–94)*	65.6 *(75–90)*	73 *(79–94)*	61 *(73–87)*	64.6 *(75–90)*	62 *(71–86)*	65.8 *(75–90)*
MCH (pg) *Reference intervals *[5]	22.8 *(26–32)*	21.3 *(26–31)*	22.1 *(26–32)*	19 *(25–30)*	21.2 *(26–32)*	20.5 *(24–29)*	20.7 *(26–31)*
HbA_0_ (%)	/	/	/	/	89	/	89.4
HbA_2_ (%) *Reference intervals*	2.9** *(1.8–3.5)*	3.8 *(1.8–3.5)*	/	4.9 *(1.8–3.5)*	4.5 *(1.8–3.5)*	3.9 *(1.8–3.5)*	3.4 *(1.8–3.5)*
HbF (%) *Reference intervals*	16.2** *(< 1)*	6.6 *(< 1)*	/	3.9 *(< 1)*	4.3 *(< 1)*	6.4 *(< 1)*	6.4 *(< 1)*
Transferrin (g/L) *Reference intervals*	/	1.64 *(2 − 3.6)*	2.1 *(2 − 3.6)*	1.94 *(2 − 3.6)*	2.16 *(2 − 3.6)*	1.91 *(2.35 − 4.03)*	/
Transferrin saturation (%) *Reference intervals*	/	8 *(16–45)*	73 *(16–45)*	39 *(16–45)*	27 *(16–45)*	36 *(16–45)*	/
Ferritin (µg/L) *Reference intervals*	/	138 *(7 − 140)*	159 *(20 − 120)*	70 *(7 − 140)*	143 *(30 − 300)*	65 *(7 − 140)*	/
Liver Iron Concentration* (mmol/kg) *Reference intervals*	310 *(3 − 33)*	19 *(3 − 33)*	48 *(3 − 33)*	22 *(3 − 33)*	/	/	38 *(3 − 33)*
Spleen craniocaudal length (cm) by MRI *Reference intervals *[6]	/	16.6 *(7.8 − 11.7)*	19.5 *(8.8 − 13.4)*	14.5 *(8 − 11.6)*	/	/	12 *(8.8 − 12)*
Spleen palpable under coastal margin (cm)	/	2	0.5	1.5	0.5	0	2
α Globin genotype	αα/αα	αα/αα		αα/αα		αα/αα	
β Globin genotype	β^A^/β^codon 97[+AC]^	β^A^/β^codon 97[+AC]^		β^A^/β^codon 97[+AC]^		β^A^/β^codon 97[+AC]^	
*BCL11A* rs1427407 G > T	/	GG		GG		GG	
*BCL11A* rs7606173 C > G	/	CG		CG		CG	
*HBG2*-polymorphism rs7482144	/	Heterozygous		Heterozygous		Heterozygous	
*HMIP* rs66650371 3 bp del	/	Wildtype		Wildtype		Wildtype	

Age corrected reference intervals, if not specified, were furnished by the laboratory of Heidelberg University Hospital,

*measured with MRI (*FerriScan*), Lusp.: Luspatercept, HU: Hydroxyurea

**these parameters were retrieved from a medical report, not directly from a laboratory report

## Discussion

Approximately 60 different mutations leading to a dominantly inherited form of β-thalassemia have been reported to online database as HbVar [7] or IthaGenes [8] and summarized by Thein [2]. Many of these mutations result in a frameshift that produces an aberrant β-globin chain, which is truncated but contains an abnormal, frequently hydrophobic, C-terminal sequence.

The heterozygous two–base pair insertion (codon 97 +AC) identified in the present family replaces the normal C-terminal 49 amino acids with a frameshifted peptide of 60 predominantly hydrophobic amino acids. The novel stop codon lies within exon 3, downstream of the original termination site, and is predicted to permit the variant mRNA to evade nonsense-mediated decay [9, 10]. Consequently, the transcript is expected to be stable and translated into an abnormal elongated β-globin chain [2]. We hypothesize that this C-terminally altered β-globin chain due to its hydrophobic properties may exert dominant-negative effects by precipitating in erythroid progenitors, leading to a clinical phenotype characterized by anemia, ineffective erythropoiesis, splenomegaly, and iron overload.

This case series illustrates the variable presentation of dominant β-thalassemia. Age at symptom onset and anemia severity differed considerably among carriers. Although the sample size precludes definitive conclusions, the pedigree suggests that male carriers of this *HBB* variant are less severely affected than female carriers: none of the suspected male carriers (II-1, II-4, III-3) required RBC transfusions, whereas four of five adult female carriers received either RBC transfusions or Luspatercept. These findings support previous observations that women with non–transfusion-dependent β-thalassemia (NTDT) are more likely than men to require occasional transfusion [11]. Alternatively, phenotypic variability may reflect unidentified genetic modifiers unlinked to the β-globin gene cluster. A possible modifier of the beta thalassemia phenotype are triplications of the alpha globin genes. As alpha globin copy numbers were not assessed, we cannot rule out that the alpha globin genotype contributed to the variability of the phenotype observed in our three patients.

The broad phenotypic spectrum observed here is consistent with previous reports of dominant β-thalassemia, such as a codon 127 (C > T) mutation producing presentations ranging from asymptomatic status to moderately severe anemia [12], and a codon 121 point mutation in Dutch families that also exhibited high variability in both sexes [13].

The late onset of symptoms in the mother underscores the importance of ongoing hematological follow-up. As in our index patients‘ mother, iron overload may result from increased intestinal absorption and should be monitored even in the absence of transfusions. Given the risk of progressive iron accumulation and secondary organ damage, regular cardiac, hepatic, and endocrine evaluations are warranted. In our series, ferritin levels in all three children were within normal limits despite the onset of hepatic siderosis. Since serum ferritin alone is insufficient to assess iron overload, MRI-based liver iron concentration measurement (e.g., *FerriScan*) should be performed when indicated.

The erythroid maturation agent Luspatercept has recently emerged as a promising therapeutic option for adults with NTDT (thalassemia intermedia) and TDT (thalassemia major) [14], capable of reducing transfusion needs and associated iron overload [15]. In both the mother and her eldest daughter, Luspatercept produced substantial hemoglobin increases, ameliorated anemia symptoms, and reduced transfusion frequency. No adverse effects were observed during the follow-up period (6–12 months), although effects on growth, endocrine status, and cardiopulmonary function could not be assessed.

Meta-analyses of TDT [16] and NTDT [17] suggest that hydroxyurea (HU) can raise total hemoglobin levels and reduce transfusion requirements. This effect was also observed in patient III-4, although the hemoglobin increase under HU was smaller than that achieved with Luspatercept, it was sufficient to improve symptoms. While long-term safety and efficacy data are limited, HU therapy to induce HbF production should be considered [18], especially in young patients, as its safety in childhood has been well established in other hemoglobinopathies such as sickle cell disease [19]. Furthermore, HU combined with erythropoietin appears to be more effective than HU alone [20]. Additional agents – including thalidomide, hepcidin agonists, and inducers – have been reported to improve ineffective erythropoiesis [21, 22] and, in combination with erythropoietin, to increase hemoglobin levels and reduce splenomegaly in murine models [23]. Improvement in hemolysis and anemia has also been demonstrated in both animal models and NTDT patients treated with the pyruvate kinase activator Mitapivat [24, 25].

In summary, we describe a novel frameshift mutation in the *HBB* gene located in a region predicted to bypass nonsense-mediated decay, thereby producing a dominantly inherited form of β-thalassemia intermedia (NTDT) in heterozygotes. The phenotypic variability within this family highlights the likely influence of additional genetic or environmental modifiers on disease severity.

## Data Availability

No datasets were generated or analysed during the current study.
